# Photobiomodulation reduces inflammation but does not influence the hypoxia-inducible factor-1α in pulp tissue of rats after bleaching

**DOI:** 10.1590/1678-7757-2021-0559

**Published:** 2022-04-08

**Authors:** Isabela Joane Prado Silva, Luciano Tavares Angelo Cintra, Edilson Ervolino, Hebertt Gonzaga dos Santos Chaves, Gustavo Sivieri-AraúJo, André Luiz Fraga Briso, Leopoldo Cosme-Silva, Francine Benetti

**Affiliations:** 1 Universidade Estadual Paulista Faculdade de Odontologia Departamento de Endodontia Araçatuba SP Brasil Universidade Estadual Paulista (UNESP), Faculdade de Odontologia, Departamento de Endodontia, Araçatuba, SP, Brasil.; 2 Universidade Estadual Paulista Faculdade de Odontologia Departamento de Ciências Básicas Araçatuba SP Brasil Universidade Estadual Paulista (UNESP), Faculdade de Odontologia, Departamento de Ciências Básicas, Araçatuba, SP, Brasil.; 3 Universidade Federal de Minas Gerais Faculdade de Odontologia Departamento de Odontologia Restauradora Belo Horizonte MG Brasil Universidade Federal de Minas Gerais (UFMG), Faculdade de Odontologia, Departamento de Odontologia Restauradora, Belo Horizonte, MG, Brasil.; 4 Universidade Estadual Paulista Faculdade de Odontologia Departamento de Odontologia Restauradora Araçatuba SP Brasil Universidade Estadual Paulista (UNESP), Faculdade de Odontologia, Departamento de Odontologia Restauradora, Araçatuba, SP, Brasil.; 5 Universidade Federal de Alagoas Faculdade de Odontologia Maceió AL Brasil Universidade Federal de Alagoas (UFAL), Faculdade de Odontologia, Maceió, AL, Brasil.

**Keywords:** Angiogenesis, Dental bleaching, Hypoxia-inducible factor-1alpha, Interleukin-23, Low-level laser therapy, Pulp inflammation

## Abstract

**Objectives:**

To evaluate the influence of photobiomodulation with infrared laser (IRL) in the rat pulp tissue after bleaching, considering the immunolabeling of interleukin (IL)-23 and hypoxia-inducible factor (HIF)-1α.

**Methodology:**

The right and left molars of forty rats were divided into groups: Control – with placebo gel and Bleached – with 35% hydrogen peroxide (H_2_O_2_). Half of the rats received one IRL application on both sides, establishing a split-mouth design, which resulted in 4 groups with 20 hemi-maxillae each: Control, Bleach, IRL, and Bleached-IRL. Rats (n=10) from each group were euthanized, at 2- and 30-days mark, and the pulp tissue was evaluated using inflammation and immunolabeling scores. Wilcoxon and Mann-Whitney statistical tests were performed (p<0.05).

**Results:**

At the 2-days mark, the Bleached group had severe inflammation and necrosis in the occlusal thirds of the pulp, and moderate to severe inflammation in cervical third, whereas the Bleached-IRL had mild to moderate inflammation (p<0.05). At the 30-days mark, there was no inflammation, but tertiary dentine formation in the bleached groups. Regarding IL-23, severe immunolabeling was observed in the Bleached group (p<0.05) at the 2-days mark; at the 30-days mark, there was a reduction in immunolabeling, in which the Bleached group had moderate and the Bleached-IRL group had mild immunolabeling (p>0.05). HIF-1α was more evident at the 2-days mark in the Bleached group, without significant difference with the Bleached-IRL (p>0.05). The difference was observed between the bleached and control groups, without immunolabeling (p<0.05); at the 30-days mark, the Bleached group had reduction in HIF-1α immunolabeling, while the Bleached-IRL had an increase; the difference remained between the bleached and the controls groups (p<0.05)

**Conclusion:**

Photobiomodulation using IRL minimized the inflammation and IL-23 immunolabeling in the pulp tissue of rats after dental bleaching, but did not influence significantly the HIF-1α immunolabeling.

## Introduction

During dental bleaching, the oxidation of organic structures of the dental tissue occurs through the action of reactive oxygen species (ROS) released from hydrogen peroxide (H_2_O_2_).^1^ Due to the ability of ROS to penetrate through mineralized dental tissues, it can reach the dental pulp^1^ and cause damage such as severe inflammation, necrosis, deposition of mineralized tissue, and pulp aging.^2^ Clinically, the response to pulp damage is presented as an intense tooth sensitivity reported by patients immediately after the bleaching.^3^

Some protocols have described methods to minimize the effects of bleaching on vital teeth, such as variations in the H_2_O_2_ concentration and duration of the bleaching gel application.^4 - 6^ Photobiomodulation therapy (PBM) was also evaluated^7 - 9^ due its biostimulant effects, as well as its analgesic and anti-inflammatory actions.^10 , 11^ Studies have shown that PBM stimulates adenosine triphosphate and protein synthesis, both capable of acting in tissue repair.^12^ Moreover, PBM increases the expression of enzymes that minimize oxidative stress in wounded rats after irradiation.^10^

Previously, it was observed that some cytokines, such as tumor necrosis factor (TNF)-α, interleukin (IL)-6 and IL-17, participate in the pulp tissue inflammatory process after bleaching.^5^ An increased concentration of H_2_O_2_ is accompanied by prolonged activation of CD5-positive cells (a receptor present in lymphocyte-like cells) even 30 days after the bleaching, when the pulp tissue is already organized.^5^ Some therapies can reduce the expression of these cytokines after bleaching.^13 , 14^

Other important cytokine previously observed in inflamed pulp tissue, the IL-23,^15^ could indicate if PBM would be able to act in this tissue. The combination IL-23 and IL-17 plays a critical role in inflammatory processes,^15 , 16^ and IL-17 was present in the pulp inflammation after dental bleaching.^5 , 13^ Thus, IL-23 may also be present in the pulp tissue of bleached teeth. PBM reduces IL-23 expression in epidermal treatment,^17^ evidencing that the laser is capable of regulating this interleukin.

ROS have also been associated with hypoxic microenvironments.^18^ The molecular response to hypoxia requires rapid action mechanisms that act within partial pressures of oxygen (O_2_), leading to the activation of transcription factors as an attempt to regulate the O_2_ supply and energy metabolism of the tissue.^18^ The transcription factor of hypoxia-inducible factor (HIF)-1α is a critical mediator of these adaptive responses and is considered the main regulator in the transition of cellular response and development of hypoxia, which induces the therapeutic properties of mesenchymal stem cells.^19^ With its interaction with genes related to angiogenesis, HIF-1α is characterized by stimulating collagen synthesis with consequent regulation of the cell cycle, favoring the survival and improvement of natural cell resistance.^20^ Recent studies have shown that HIF-1α promotes the expression of IL-1β and TNF-α in dental pulp cells, suggesting that HIF-1α is involved in the progress of inflammation in dental pulp.^19^

Angiogenesis is the formation of new capillaries and PBM has an important effect on this process.^21 , 22^ Transient hyperemia in the pulp was observed after PBM application in teeth during orthodontic movement and PBM was related to accelerated pulp tissue repair.^21^ It was also observed that angiogenesis on dorsal wound of rats was more intense after treatment with PBM.^22^ Although vascular permeability is increased in pulp tissue of bleached rat teeth,^23^ the angiogenesis process in these teeth has not yet been evaluated.

Thus, the aim of this study is to evaluate the influence of PBM on pulp inflammation of bleached teeth through the analysis of the inflammatory infiltrate and the immunolabeling of pro-inflammatory cytokine IL-23. Furthermore, this study evaluated the presence of HIF-1α in the pulp tissue of these teeth, and the influence of PBM on the immunolabeling of this transcription factor. The null hypothesis adopted was that PBM does not influence the inflammatory process and the HIF-1α immunolabeling in the pulp tissue of bleached teeth.

## Methodology

### Experimental design

Forty 2-month-old male Wistar rats (weighing approximately 280 g) were used. The sample size was based on the findings of previous studies.^2^ The animals were maintained in a temperature-controlled environment (22°C±1°C, 70% humidity, and a 12-h light–dark cycle) and received water and food *ad libitum* . All animal procedures were performed in accordance with the Guide for the Care and Use of Laboratory Animals of the National Institutes of Health (Bethesda, MD, USA). The experimental protocol (CEUA-00713) was approved by the local Ethics Committee.

### Dental bleaching and PBM treatment

The animals were anaesthetized by intramuscular injections of ketamine (80 mg/kg, Ketamina Agener 10%; União Química Farmacêutica Nacional S/A, Embu-Guaçu, São Paulo, Brazil) and xylazine (10 mg/kg, Xilazin; Syntec do Brasil LTDA, Cotia, São Paulo, Brazil). After the application and photo-activation of the resinous gingival barrier (Top Dam; FGM Dental Products, Joinville, SC, Brazil), the right and left maxillary molars of the rats were each randomly treated with either bleaching gel (0.01 mL, single application of 30 min of the 35% H_2_O_2_; Whiteness HP Maxx, FGM Dental Products, Joinville, SC, Brazil) or placebo gel (0.01 mL, single application of 30 min of the thickener of the bleaching gel).^4^ The molars of each rat were randomized by lottery for application of treatment. After bleaching, the tooth surface was wiped with cotton and absorbent paper and rinsed thoroughly with water. To standardize the applied volume of the bleaching gel, 1.0 mL syringes were used.^5^

PBM was performed in half of the animals according Terayama, et al.^14^ (2020) and according to the protocol suggested by the manufacturer for the treatment of dental hypersensitivity. A duo semiconductor infrared laser (IRL) (GaA1As; MM Optics Ltda, São Carlos, SP, Brazil) with a wavelength of 808 nm, was applied immediately after bleaching, for 30 seconds. The device had a fixed power of 100 mW. The beam exit area was of 3 mm,^2^ the tip of the laser pen was kept in contact with the first molar^7 , 11^ .

**Figure 1:**
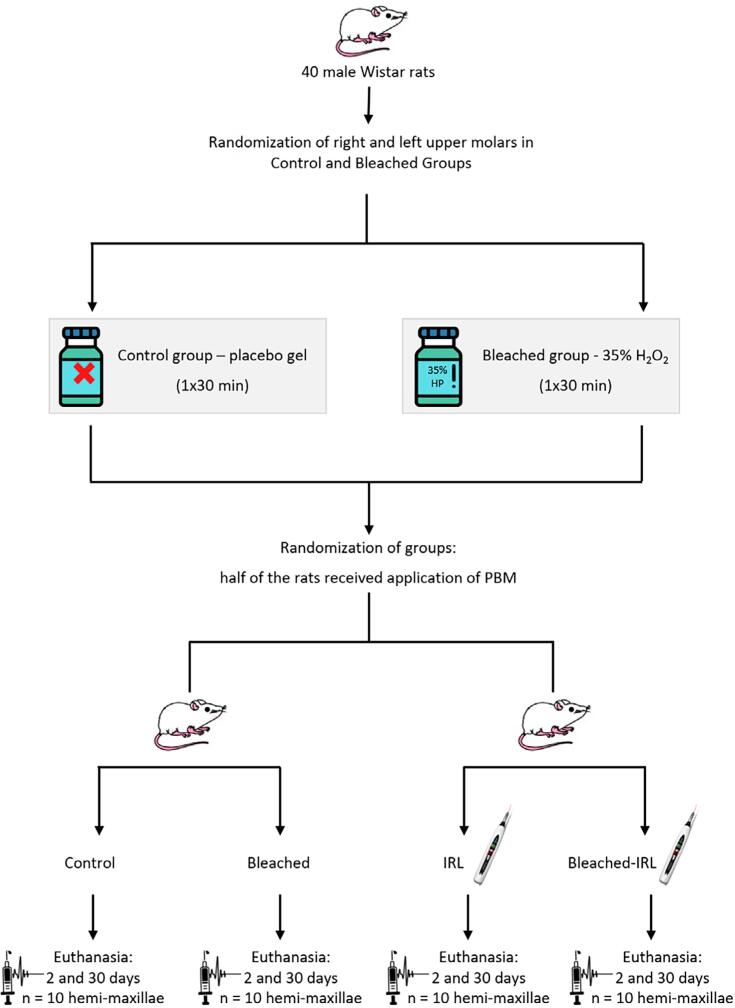
Diagram showing the distribution and randomization of rats in each group

The split-mouth design was established after the bleaching and PBM treatments, resulting in 4 experimental groups with 20 hemi-maxillae each: Control, bleached, IRL, and bleached-IRL. Figure 1 shows the division of groups.

### Histological analysis

At 2 days and at 30 days,^5^ the animals were euthanized (a total of 20 rats per period, resulting in 40 molars per period; n=10 per group) with an overdose of sodium thiopental (150 mg/kg; Thipentax, Cristália, Produtos Químicos Farmacêuticos LTDA, Itapira, SP, Brazil). The hemi-maxillae were separated, dissected, and fixed in a solution of 4% buffered formaldehyde (24 hours). The specimens were decalcified in a 10% ethylenediaminetetraacetic acid (3 months) and then dehydrated, clarified, and embedded in paraffin. Serial histological sections of each specimen were selected from the point where the mesial root of the first molar was at its full longitudinal extension. Subsequently, 5-µm sections were cut in the buccolingual plane. The blades with histological sections were then stained with hematoxylin-eosin for analysis using light microscopy (×400 magnification; DM4000 B; Leica Microsystems, Wetzlar, Germany). For each specimen, 2 slides were used with 3 tissue sections each. The pulp chamber was divided by occlusal, middle, and cervical thirds^24^ and the analysis of the inflammatory infiltrate was scored ( Table 1 ).^2^ The analysis was performed by a single calibrated operator in a blinded manner.

**Table 1:** Scores attributed to the intensity of inflammatory infiltrate and immunolabeling

Score	Inflammatory infiltrate
0	Inflammatory cells absent or negligible in number
1	Mild inflammatory infiltrate (<25 cells per field)
2	Moderate inflammatory infiltrate (between 25 and 125 cells per field)
3	Severe inflammatory infiltrate (>125 cells per field)
4	Tissue necrosis
**Score**	Immunolabeling pattern
0	Immunolabeling missing (absence of labelling in ECM and absence of immunoreactive cells)
1	Low pattern of immunolabeling (weak labelling of the ECM and approximately ¼ of the immunoreactive cells)
2	Moderate pattern of immunolabeling (moderate labelling of the ECM and approximately ½ of the immunoreactive cells)
3	Severe pattern of immunolabeling (strong labelling of the ECM and approximately ¾ of the immunoreactive cells)
4	Very severe pattern of immunolabeling (extremely strong labelling of the ECM and approximately all immunoreactive cells)

ECM: extracellular matrix.

### Immunohistochemical analyses

Other histological sections of groups were obtained for immunohistochemical assessments with an indirect immunoperoxidase technique^13^ for IL-23 and HIF-1α. The histological sections were deparaffinized in xylene and hydrated in a decreasing ethanol series. Antigen retrieval was performed by immersing the histological slides in buffer citrate solution (Antigen Retrieval Buffer; Spring Bioscience, Pleasanton, CA, USA) in a pressurized chamber (Decloaking Chamber; Biocare Medical, Concord, CA, USA) at 95°C for 10 min. The slides were rinsed with phosphate-buffered saline at the end of each stage of the immunohistochemical reaction. The histological sections were immersed in 3% H_2_O_2_ solution for 1 h and 20 min and in 1% bovine serum albumin for 12 h to block the endogenous peroxidase activity and nonspecific sites, respectively. The histological slides were divided and incubated with one of the following primary antibodies: anti-HIF-1α and anti-IL-23 (rabbit primary antibodies; Sigma-Aldrich Co. LLC, St. Louis, MO, USA). The primary antibodies were diluted (Antibody Diluent with Background Reducing Components; Dako Laboratories, Carpinteria, CA, USA) and placed in a moist chamber for 24 h. The histological sections were then incubated with a biotinylated secondary antibody for 1 h and 30 min and subsequently treated with streptavidin–horseradish peroxidase conjugate for 1 h and 30 min (Universal Dako Labelled Streptavidin-Biotin kit; Dako Laboratories). After rinsing with PBS, the reaction was developed using the chromogen 3,3’-diaminobenzidine tetrahydrochloride (DAB Chromogen kit; Dako Laboratories) and counterstained with hematoxylin. The negative controls consisted of specimens that underwent the previously described procedures without treatment with the primary antibodies.

For the analyses, the immunolabelling was defined as the presence of a brownish color in the cytoplasm of the cells and extracellular matrix. A semi-quantitative analysis was performed, which provides information on the numbers of immunolabeled cells and immunolabelling intensity of the extracellular matrix.^2^ Table 1 shows the scores .^2^ Data were collected and analyzed by a single-calibrated and blinded operator.

### Statistical analyses

The Wilcoxon signed-rank and the Mann-Whitney test were used for statistical comparisons of pulp damage, inflammatory response, and immunohistochemistry at the significance level of 5%.

## Results

### Analysis of the inflammatory infiltrate

**Figure 2:**
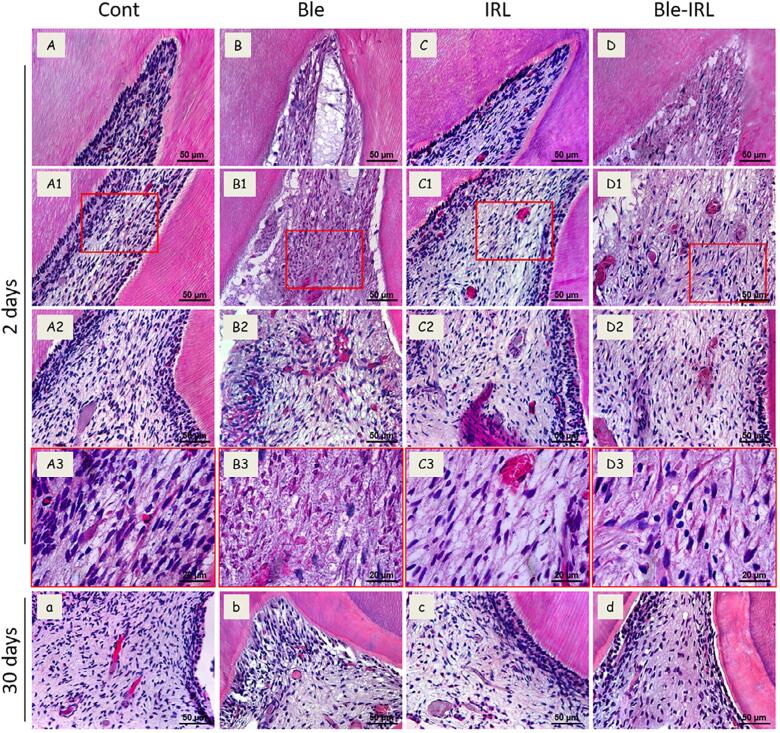
Representative images of the inflammation analysis of the groups at 2 and 30 days. Images of (A-D) occlusal, (A1-D1) middle, and (A2-D2) cervical thirds of groups, and (A3-D3) higher magnification to evidencing pulp tissue of each group, at 2 days. (A-A3) Control group without alteration and absence of inflammation; (B-B3) Bleached group with evident necrosis area and severe inflammatory infiltrate; (C-C3) IRL group with organized pulp tissue and absence of inflammation; and (D-D3) Bleached-IRL group with moderate to mild inflammation. (a-d) Images of (a) control, (b) Bleached, (c) IRL and (d) Bleached-IRL groups at 30 days, evidencing absence of inflammation and presence of tertiary dentine in bleached groups. [400×, 1000×; H.E.]

Figure 2 shows the representative images of the inflammatory infiltrate analysis at 2 and 30 days, and Table 2 shows the results . At the 2-days mark, it was possible to observe a higher number of inflammatory cells and the presence of necrosis in the occlusal and middle thirds of the pulp tissue in the Bleached group when compared to the Bleached-IRL group, which showed lesser inflammatory infiltrate and absence of necrosis ( *p* <0.05). In the cervical third of the teeth, there was moderate to severe inflammatory infiltrate in the Bleached group, and mild inflammatory infiltrate in the Bleached-IRL group, with a significant difference between both groups ( *p* <0.05).

**Table 2:** Median of scores attributed to inflammatory infiltrate and to immunolabeling of IL-23 and HIF-1α in each group

Analysis	Scores		Groups				P value
		Cont	Ble	IRL	Ble-IRL		
HE - 2 days	Occlusal	Median	0	4	0	2	* Cont×Ble: =0.002 *IRL×Ble-IRL: =0.002 * Ble×Ble-IRL: <0.001
	Middle	Median	0	3	0	2	* Cont×Ble: =0.002 * IRL×Ble-IRL: =0.002 * Ble×Ble-IRL: <0.001
	Cervical	Median	0	2	0	1	* Cont×Ble: =0.002 IRL×Ble-IRL: =0.063 * Ble×Ble-IRL: <0.001
IL-23	2 days	Median	0	3	0	2	* Cont×Ble: =0.002 * IRL×Ble-IRL: =0.016 * Ble×Ble-IRL: <0.001 Cont×IRL: =0.335
	30 days	Median	0	2	0	1	* Cont×Ble: =0.004 * IRL×Ble-IRL: =0.016 Ble×Ble-IRL: =0.257 Cont×IRL: =0.681
HIF-1α	2 days	Median	0	3	0	2	* Cont×Ble: =0.002 * IRL×Ble-IRL: =0.002 Ble×Ble-IRL: =0.089 Cont×IRL: =0.681
	30 days	Median	0	2	0	3	* Cont×Ble: = 0.002 * IRL×Ble-IRL: = 0.002 Ble×Ble-IRL: = 0.185 Cont×IRL: = 0.185

Cont×Ble and IRL×Ble-IRL = Wilcoxon test (P<0.05); Ble×Ble-IRL and Cont×IRL = Mann-Whitney test (P<0.05).

* The symbol highlights the significant difference among groups. Con.: Control; Ble: Bleached; IRL: infrared laser.

The inflammation and necrosis observed in Bleached group was significantly different compared to the control group, which showed no inflammation in all thirds of the pulp chamber ( *p* <0.05). In contrast, the inflammation observed in the Bleached-IRL group was significantly different compared to the IRL group, which showed no inflammation, only in the occlusal and middle thirds ( *p* <0.05); in the cervical third, there was no significant difference between the Bleached-IRL and IRL groups ( *p* >0.05).

At 30 days, all groups had pulp tissue with cellular organization, presence of odontoblastic layer, and absence of inflammatory cells. Both bleached groups had an extensive deposition of tertiary dentine ( Figure 2 ; Table 2 ).

### Immunohistochemical analyses

**Figure 3:**
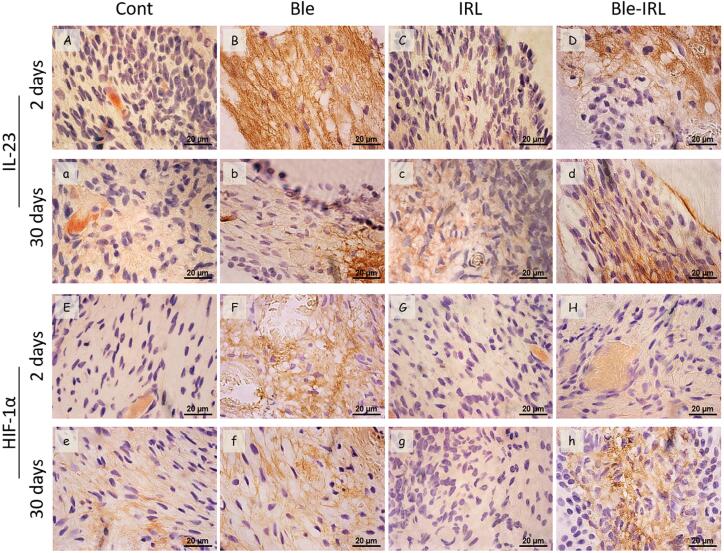
Representative images of the immunolabeling of IL-23 and HIF-1α for each group, at 2 and 30 days. Images of (A,a) control and (C,c) IRL groups with absence of IL-23 immunolabeling at (A, C) 2 and (a, c) 30 days; (B,b) Bleached group with severe and moderate immunolabeling at (B) 2 and (b) 30 days, respectively; and (D,d) Bleached-IRL group with moderate and mild immunolabeling at (D) 2 and (d) 30 days, respectively. Images of (E,e) control and (G,g) IRL groups with absence of HIF-1α immunolabeling at (E, G) 2 and (e, g) 30 days; (F,f) Bleached group with moderate and mild immunolabeling at (F) 2 and (f) 30 days, respectively; and (H,h) Bleached-IRL group with mild and moderate immunolabeling at (H) 2 and (h) 30 days, respectively. [1000×; immunohistochemical]

Figure 3 shows the representative images of the immunohistochemical analyses and Table 2 shows the results. Regarding the immunolabeling of IL-23 at the 2-days mark, a higher immunolabeling was observed in the Bleached group when compared to the Control group, which showed no immunolabeling in most specimens ( *p* <0.05). Moreover, the Bleached group differed significantly from the Bleached-IRL group, which had moderate immunolabeling ( *p* <0.05). The IRL group had no immunolabeling, unlike the Bleached-IRL group ( *p* <0.05), and was similar to the control group ( *p* >0.05).

At the 30 days mark, there was a reduction in IL-23 immunolabeling in the bleached groups, in which the Bleached group had moderate immunolabeling and the Bleached-IRL group had mild immunolabeling, but with no significant difference between both ( *p* >0.05). The difference remained between the bleached groups and their respective controls ( *p* <0.05).

Regarding the immunohistochemical analysis of HIF-1α, the immunolabeling was more evident at the 2-days mark in the Bleached group, with a severe immunolabeling in most specimens, whereas the Bleached-IRL group had moderate immunolabeling; however, there was no significant difference between the groups ( *p* >0.05). The difference was observed between the bleached groups and their respective controls, which had no immunolabeling ( *p* <0.05). At the 30-days mark, the Bleached group had a reduction in HIF-1α immunolabeling, whereas the Bleached-IRL group had an increase in immunolabeling from moderate to severe; however, the difference remained only between the bleached groups and their controls ( *p* >0.05) ( Figure 3 ; Table 2 ).

## Discussion

This study evaluated, *in vivo* , the influence of PBM applied on the inflammatory process following dental bleaching, using the immunolabeling of IL-23 cytokine and the angiogenesis marker (HIF-1α). We observed a significant reduction in the inflammatory infiltrate and in the presence of necrosis in the group that received PBM compared with the group that did not receive it. We also observed a reduction from severe IL-23 immunolabeling in the Bleached group to moderate immunolabeling in the Bleached-IRL group. However, the immunolabeling of HIF-1α did not differ significantly between the bleached groups, regardless of the application of PBM. Thus, the null hypothesis that PBM does not influence the inflammation in the pulp of bleached teeth was rejected, but the null hypothesis that PBM does not influence the HIF-1α immunolabeling in pulp after bleaching was accepted.

A previous study compared the action of red laser (RL) and IRL on the pulp tissue of rat molars after bleaching and observed similar effects of reduced inflammatory infiltrate with a single application of IRL as well as with three applications of RL.^14^ Our study used only a single application of the IRL, due to reduction of inflammation on the dental pulp and shorter clinical time. Another study that evaluated the potential of PBM in minimizing the damage caused by dental bleaching to pulp cells showed that the further reduction in the cytotoxicity of the bleaching gel was obtained using IRL compared to RL^7^ , corroborating our results.

Studies have shown that the beneficial effects of PBM are related to its ability to stimulate cell proliferation, which was observed in pulp cells after stimulation.^25 , 26^ Induction of gene expression of these cells and stimulation of dentine production was also observed.^26^ In pulpotomy, PBM stimulated the repair and promoted the healing of the pulp tissue,^27^ and presented anti-inflammatory potential.^28^ Moreover, PBM favored the reduction of the exudative phase of the inflammatory process and promoted an increase in vascularization and collagen synthesis.^29^ These studies showed the beneficial effects of PBM for the repair of pulp tissue, which corroborates our results.

Previously, an increased expression of pro-inflammatory cytokines was observed in the pulp tissue of bleached teeth.^5 , 13^ The production of IL-23 cytokine is related to the formation of determined cell types, such as antigen-presenting cells, monocytes, activated dendritic cells, and macrophages.^30^ It has been shown that IL-23 is important and necessary for the expansion and stabilization of cells in chronic inflammatory reactions.^31^ Thus, the reduction in the immunolabeling of this cytokine would be a beneficial indicator of the effects of different therapies.

A previous study, which evaluated the action of PBM in the treatment of cutaneous inflammation, showed that laser application suppressed the expression of IL-23.^17^ Our study showed that the IRL was able to reduce significantly the immunolabeling of IL-23, being an indicator of the effectiveness of this therapy in reducing the damage caused by the bleaching gel to the pulp tissue.

Both the expression of pro-inflammatory cytokines and hypoxia are factors directly related to tissue inflammation at the molecular level.^32^ In these cases, the stabilization of HIF-1α is boosted, promoting cell adaptation to the hypoxic environment.^33^ HIF-1α is intended to promote angiogenesis to meet the need for oxygen, and it was found in endothelial, osteoblasts, and inflammatory cells.^34^ Moreover, it was activated during active inflammation by metabolic shifts toward hypoxia.^33^

HIF-1α plays an important role in survival and improving cell resistance during the inflammatory process. A previous study showed that overexpression of HIF-1α improves the immunomodulation of dental mesenchymal stem cells (MSCs).^35^ It was proposed that the expression of this marker by MSCs would be beneficial for the resolution of the inflammatory process by inducing the recruitment and differentiation of suppressor macrophages.^35^ Moreover, the MSCs with HIF-1α expression exhibited greater resistance to natural killer cell-mediated lysis, resulting in reduced MSCs death.^35^ These data show the importance of the presence of HIF-1α in tissue inflammation.

It was demonstrated that PBM could modulate HIF-1α, promoting a therapeutic approach by reduce hypoxia, which resulted in a reduced expression of this marker in macrophages that coordinate the inflammatory process.^36^ However, PBM increased the immunolabeling of HIF-1α in cancerous tissue according to the number of applications of PBM.^37^ This may be due to the induction of angiogenesis that PBM can provide to the tissue.^22^ PBM also promoted bone regeneration, with an increase in related gene expression, including HIF-1α; according to the authors, cells exposed to laser radiation undergo a sustained increase in ROS production through the alteration of the mitochondrial membrane potential, which further amplifies the induction of HIF-1α.^38^ Thus, it was expected that PBM would lead to a significant increase in HIF-1α immunolabeling in our study.

We observed that the pulp tissue of bleached teeth had a higher immunolabeling for HIF-1α. It is known that cells exposed to hypoxia have higher levels of HIF-1α than normal cells,^39^ which corroborates the results found in this study, since H_2_O_2_ promotes increased oxidative stress on the pulp tissue^1^ , similar to tissues in cancerous environment.^37^ However, the present data showed that PBM was not able to significantly change the immunolabeling of HIF-1α after damages caused by H_2_O_2_ to the pulp tissue, despite the later increase in the immunolabeling of this marker. Previously, it was observed that ROS act in the stabilization of HIF-1α,^40^ which may explain the results of this study. To our knowledge, there are no other studies that have evaluated the influence of PBM on HIF-1α modulation in pulp tissue after bleaching. Considering that the effects of PBM in various treatments are still questionable,^9 , 37^ further studies should be conducted to determine an ideal protocol, according to each clinical indication.

Considering the data observed in this and previous studies,^7 , 14^ we can suggest that PBM represents satisfactory results in minimizing inflammatory processes in pulp tissue. However, PBM was not able to allow the recovery of a pulp tissue without changes, even 30 days after the bleaching, since there was a significant difference compared to its control group. Moreover, this study highlights that the H_2_O_2_ effects in pulp tissue are so intense that they can prevent the full action of therapies, such as the PBM. This reinforces the importance of using low concentrations of H_2_O_2_ in the bleaching gel.^1 , 5^

## Conclusion

The PBM, with IRL, minimized the inflammatory infiltrate and IL-23 immunolabeling in the pulp tissue of rats after dental bleaching, but did not significantly influence HIF-1α immunolabeling.
